# Posterior Vitreous Detachment Precipitated by Yoga

**DOI:** 10.7759/cureus.2109

**Published:** 2018-01-24

**Authors:** Soh Yee Chong, Lai Chan Fhun, Evelyn Tai, Mei Fong Chong, Khairy Shamel Sonny Teo

**Affiliations:** 1 Ophthalmology, School of Medical Sciences, Universiti Sains Malaysia, 16150 Kubang Kerian, Kelantan, Malaysia; 2 Ophthalmology, Hospital Raja Permaisuri Bainun, Ipoh

**Keywords:** yoga, posterior vitreous detachment, vitreous hemorrhage

## Abstract

Yoga has recently been touted as a means to improve physical and mental well-being. However, no form of exercise is without its risks. A 32-year-old Chinese female with moderate myopia complained of right eye sudden onset of floaters and mild blurring of vision after the head-down posture. The visual acuity was 6/12 in the right eye and 6/9 in the left eye. A right eye fundus examination showed posterior vitreous detachment, with a small blood clot located at the inferior margin of the optic disc. The patient was diagnosed with right eye vitreous hemorrhage secondary to acute posterior vitreous detachment and was managed conservatively. Acute changes in posture, especially between an upright and a head-down position, may cause acute posterior vitreous detachment. As yoga practitioners may be required to assume this head-down position, myopic patients should be warned of the possible ocular complications of this exercise.

## Introduction

Yoga is an ancient form of exercise that focuses on strength, flexibility, and breathing, to promote physical and mental well-being. Yoga has been found to be beneficial in various conditions and can reduce cardiovascular risk and improve general mental well-being [1-2]. On the other hand, yoga has its risks. We report an unusual case of vitreous hemorrhage secondary to posterior vitreous detachment after yoga.

## Case presentation

A 32-year-old Chinese female with moderate myopia (-4.00 D OU) presented to us, complaining of floaters over the right eye after the head-down posture during yoga. The position was sustained for three minutes, and she noticed the floaters approximately 15 minutes later, associated with a mild blurring of vision. There were no flashes, visual field defects, or recent history of ocular trauma. The patient had no symptoms of bleeding tendencies nor was she on antiplatelet or anticoagulant therapy.

**Figure 1:**
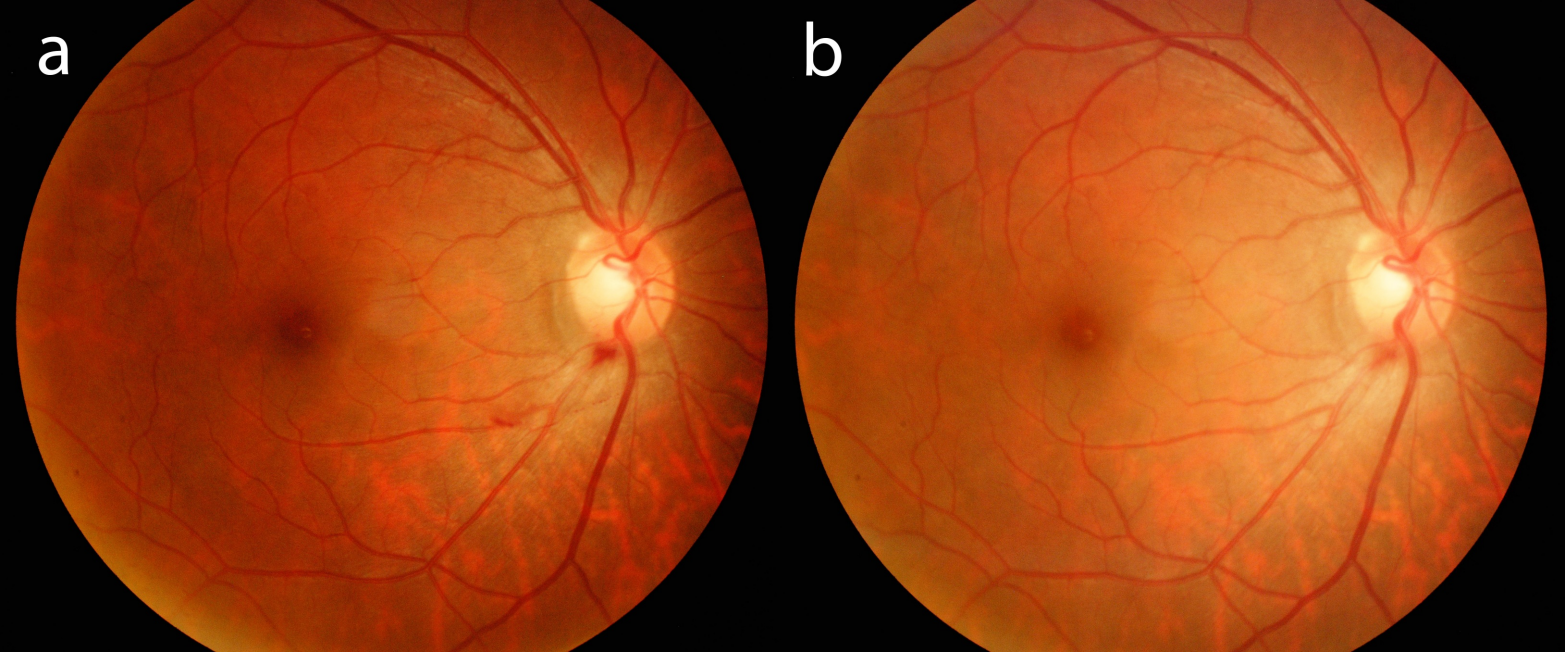
Right eye fundus photo on presentation and three months later

Her visual acuity was 6/12 in the right eye and 6/9 in the left eye. The anterior segment examination was unremarkable bilaterally. The right eye fundus examination showed posterior vitreous detachment with a small blood clot located at the inferior margin of the optic disc (Figure 1a). No retinal breaks or tears were seen. The left fundus was normal. A systemic examination did not reveal any sign of injury or bleeding tendency. The full blood count and coagulation profile were normal. The patient was diagnosed with right eye vitreous hemorrhage secondary to acute posterior vitreous detachment. She was managed conservatively. Three months later, the floaters and blurring of vision resolved. Her right eye visual acuity improved to 6/9. The blood clot in her right eye had contracted (Figure 1b).

## Discussion

Yoga has become increasingly popular. In one report, the prevalence of yoga practice doubled within a 10-year period, from 0.46% in 1999 to 1.11% in 2008 [3]. Yoga has been advocated to reduce stress, enhance mental well-being, and improve the quality of life [1].

Posterior vitreous detachment is a common consequence of aging due to vitreous liquefaction, resulting in the separation of the vitreous from the retina. It commonly occurs in people older than 60 years of age. However, certain conditions, such as trauma, myopia, cataract surgery, and inflammation, may accelerate the development of posterior vitreous detachment. In our case, the patient was myopic; these patients have been shown to have an altered composition of their vitreous, which may predispose them to vitreous degeneration or syneresis [4].

During yoga exercises, postural changes, especially from an upright position to a head-down position and vice versa, may cause abrupt shifts in the vitreous gel. This process may accelerate the process of posterior vitreous detachment, particularly in a syneretic eye. Vitreous hemorrhage may be seen in acute posterior vitreous detachment, due to the spontaneous rupture of small retinal capillaries. The treatment of this type of hemorrhage is usually conservative.

Another potential complication of acute posterior vitreous detachment is a retinal break or retinal tear, which can subsequently lead to rhegmatogenous retinal detachment [5]. For this reason, patients are usually discouraged from straining or heavy exercise for at least s weeks after an acute posterior vitreous detachment.

In our review of the literature, we found various other case reports related to the ocular adverse effects of yoga. The head-down body position ("sirsasana”) has been observed to cause an increase in intraocular pressure and a worsening of glaucomatous visual field defects [6]. In a recent case report, the increased intraocular pressure induced by this position has also been associated with branch retinal vein occlusion [7]. In an interview-based study of 76 yoga practitioners, approximately 11.8% had ocular complications, such as acute glaucoma, worsening of chronic glaucoma, orbital varices, and retinal vein occlusion [8]. Thus, patients with glaucoma, myopia, and those with hypertension or other risk factors for retinal vein occlusion should be counseled regarding the health risks of yoga before joining a class. We recommend that these patients avoid exercises involving the head-down position.

## Conclusions

Acute changes in posture, especially between an upright position and a head-down position, may cause acute posterior vitreous detachment. As yoga practitioners may be required to assume the head-down position, also known as "sirsasana," patients with myopia or ocular abnormalities should be warned of the possible ophthalmological complications of this exercise.

## References

[1] Vancampfort D, Vansteelandt K, Scheewe T, Knapen PJ, De Herdt A, De Hert M (2012). Yoga in schizophrenia: a systematic review of randomised controlled trials. Acta Psychiatr Scand.

[2] Innes KE, Bourguignon C, Taylor AG (2005). Risk indices associated with the insulin resistance syndrome, cardiovascular disease, and possible protection with yoga: a systematic review. J Am Board Fam Pract.

[3] Ding D, Stamatakis E (2014). Yoga practice in England 1997-2008: prevalence, temporal trends, and correlates of participation. BMC Res Notes.

[4] Itakura H, Kishi S, Li D, Nitta K, Akiyama H (2014). Vitreous changes in high myopia observed by swept-source optical coherence tomography. Invest Ophthalmol Vis Sci.

[5] Takkar B, Azad S, Shashni A, Pujari A, Bhatia I, Azad R (2016). Missed retinal breaks in rhegmatogenous retinal detachment. Int J Ophthalmol.

[6] Bertschinger DR, Mendrinos E, Dosso A (2007). Yoga can be dangerous —glaucomatous visual field defect worsening due to postural yoga. Br J Ophthalmol.

[7] Balamurugan A, Srikanth K (2016). A rare case of branch retinal vein occlusion following sirsasana. Int J Yoga.

[8] Cramer H, Krucoff C, Dobos G (2013). Adverse events associated with yoga: a systematic review of published case reports and case series. PLoS One.

